# Molecular Design of Soluble Biopolyimide with High Rigidity

**DOI:** 10.3390/polym10040368

**Published:** 2018-03-26

**Authors:** Sumant Dwivedi, Tatsuo Kaneko

**Affiliations:** 1 Graduate School of Advanced Science and Technology, Energy and Environment Area, Japan Advanced Institute of Science and Technology, 1-1 Asahidai, Nomi, Ishikawa 923-1292, Japan; supernalsumant@gmail.com; 2 Japan Science and Technology, ALCA, Tokyo 102-0076, Japan

**Keywords:** biopolyimide, soluble polymers, torsion energy, solubility trend

## Abstract

New soluble biopolyimides were prepared from a diamine derived from an exotic amino acid (4-aminocinnamic acid) with several kinds of tetracarboxylic dianhydride. The biopolyimide molecular structural flexibility was tailored by modifying the tetracarboxylic dianhydride moiety. The obtained polyimides were soluble in various solvents such as *N*-methyl-2-pyrrolidone, *N*,*N*-dimethylacetamide, *N*,*N*-dimethylformamide, dimethyl sulfoxide, and even tetrahydrofuran. It was observed that the biopolyimide solubility was greatly dependent upon the structural flexibility (torsion energy). Flexible structure facilitated greater solubility. The synthesized biopolyimides were largely amorphous and had number-average molecular weight (*M*_n_) in the range (5–8) × 10^5^. The glass transition temperatures (*T*_g_) of the polymers ranged from 259–294 °C. These polymers exhibited good thermal stability without significant weight loss up to 410 °C. The temperatures at 10% weight loss (*T*_d10_) for synthesized biopolyimide ranged from 375–397 °C.

## 1. Introduction

Aromatic polyimides (PI) have gained a reputation for exhibiting outstanding thermal behavior and have been used in a wide range of applications such as electronics, coatings, composite materials, linear and non-linear optical materials, and membranes [1,2,3,4]. However, the commercial use of these materials is often limited because of the difficult and expensive processing owing to their poor solubility and high softening or melting temperatures. To resolve these problems, many researchers have focused on synthesizing soluble and processable aromatic polyimides in a fully imidized form without compromising their excellent physical properties. Several synthetic modifications of basic rigid-chain structures, including the introduction of flexible linkages, bulky side substitutions, and asymmetric monomers into the backbone, have been attempted [5,6,7,8]. 

Soluble polyimides, such as ULTEM 1000 resin, are commercially available but possess low softening temperature (217 °C) accompanied by rapid thermal degradation (171 °C) [9]. The conventional methodology for improving solubility is to introduce a bulkier side chain of aromatic polyimides, which probably decreases inter-chain interactions and thereby reduces the rigidity of the polymer [10,11,12,13]. For example, Yagci and Mathias reported several kinds of polyimides synthesized from trimethyl and di-*tert*-butylhydroquinone-based ether-linked diamines [14]. Hsiao et al. synthesized an aromatic polyimide based on 1,2-bis(4-animophenoxy)-4-*tert*-butylbenzene [15]. Liaw et al. prepared some polyimides from 1,4-bis(4-aminophenoxy)-2-*tert*-butylbenzene [16]. Langsam and Burgoyne investigated the gas permeability of some polyimides synthesized from bridged diamines containing *tert*-butyl groups and 5,5′-[2,2,2-trifluoro-1-(trifluoromethyl)ethylidene]bis-1,3-isobenzofuranedione by a two-step traditional method [17]. Many researchers have also tried random monomer combinations to evaluate the synthesized polyimide solubility and its physical characteristics [7,18]. Furthermore, a molecular design strategy has been utilized to establish the empirical relationship for amorphous polymer solubility behavior, especially those which exhibit glass-rubber transition phenomenon. It was found that the polymer solubility behavior was greatly dependent on molecule specific solvent-interactive volume and cohesive energy [19,20]. Determination of the solubility trend for all random monomer combinations from experimental methods is time consuming and labor intensive [18]. Moreover, the established molecular design methodologies are either too complex to be applied practically or focused on compromising the rigid chain polymer backbone by introducing bulkier units. Specifically, the conventional molecular designing methodologies lack the systematic relationship between the aromatic polyimide structural characteristics and their solubility behavior [20]. Recently, a biologically existing exotic amino acid, 4-aminocinnamic acid (4ACA), was discovered and used to prepare an aromatic diamine for biopolyimide synthesis [21,22]. This article describes the synthesis of a series of biopolyimides using 4ACA derived diamine with various kinds of dianhydride in a two-step process. The systematic molecular design enabled biopolyimide structural features correlation with the solvent specific solubility behavior at room temperature.

## 2. Experimental

### 2.1. Materials 

Dianhydride, 1,2,4,5-cyclohexanetetracarboxylic dianhydride (CHDA), 1,2,3,4-cyclopentanetetracarboxylic dianhydride (CPDA) and *meso*-butane-1,2,3,4-tetracarboxylic dianhydride (MSDA, TCI) were purified by sublimation under reduced pressure before use. Diamine precursor 4-aminocinnamic acid (4ACA, Tokyo Chemical Industries, Tokyo, Japan), esterification reagent trimethylsilyl chloride (TMSCl, Sigma-Aldrich, Tokyo, Japan), and polymerization solvent super-dehydrated *N*,*N*-dimethylacetamide (DMAc, Kanto Chemical Corporation, Tokyo, Japan) and solvents were used as received. *N*-Methyl-2-pyrrolidone (NMP), dimethyl sulfoxide (DMSO), *N*,*N*-dimethylformamide (DMF), tetrahydrofuran (THF), and other chemicals were of research grade and used as received. 

### 2.2. Syntheses 

The monomer and biopolyimide syntheses procedure are shown in the Scheme 1 and Figure 1, respectively. The characterization of the monomer and biopolyimide has been described in the Supporting Information (Figures S1–S6). 

### 2.3. Characterization 

The number-average molecular weight (*M*_n_), weight-average molecular weight (*M*_w_), and the molecular weight distribution (PDI) were determined by gel permeation chromatography (GPC, concentration 5 g/L, DMF eluent, Shodex SB-800HQ, Showa Denko K.K., Tokyo, Japan) after calibration with pullulan standards. 

NMR measurements were performed by a Bruker Biospin AG 400 MHz, 54 mm spectrometer using DMSO-d_6_ as the solvent. The FT-IR spectra were recorded with a Perkin-Elmer Spectrum One spectrometer between 4000 and 600 cm^−1^.

Thermogravimetric analysis (TGA) and differential scanning calorimetry (DSC) were performed on Seiko Instruments SII, SSC/5200 and Seiko Instruments SII, X-DSC7000T, respectively, at a heating rate of 10 °C/min under nitrogen atmosphere. The polymer specimens were dried at 100 °C for 1 h to remove any absorbed moisture before both TGA as well as DSC measurements. 

The ultraviolet-visible (UV-Vis) optical absorption spectra were recorded on a Perkin-Elmer, Lambda 25 UV/vis spectrophotometer at room temperature over the range of 200–800 nm. X-ray diffraction (XRD, Rigaku SmartLab, Tokyo, Japan) pattern was used to determine the crystallinity degree of the polyimide films. 

Polyimide solubility was checked at room temperature in various polar (protic and aprotic) and non-polar solvents with a concentration of (10 mg/mL). Chem3D^®^Pro14.0.0.117 version was used to determine the torsion energy and the polar surface area. Torsion energy was determined by minimizing energy at 298 K.

## 3. Results and Discussion

### Biopolyimide Synthesis and Structural Characteristics 

**Figure 1:**
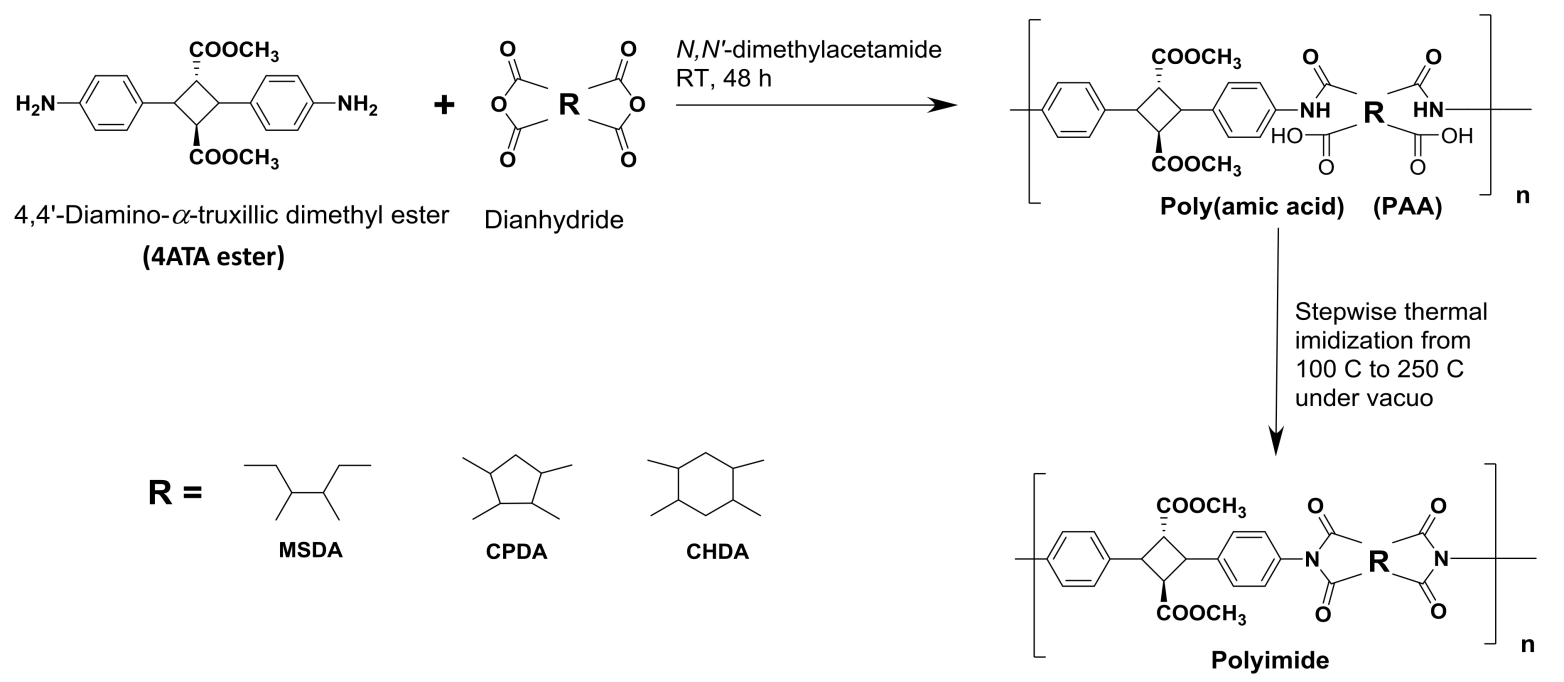
Polymerization scheme between 4,4′-Diamino-α-truxillic dimethyl ester and various dianhydrides.

**Figure 2:**
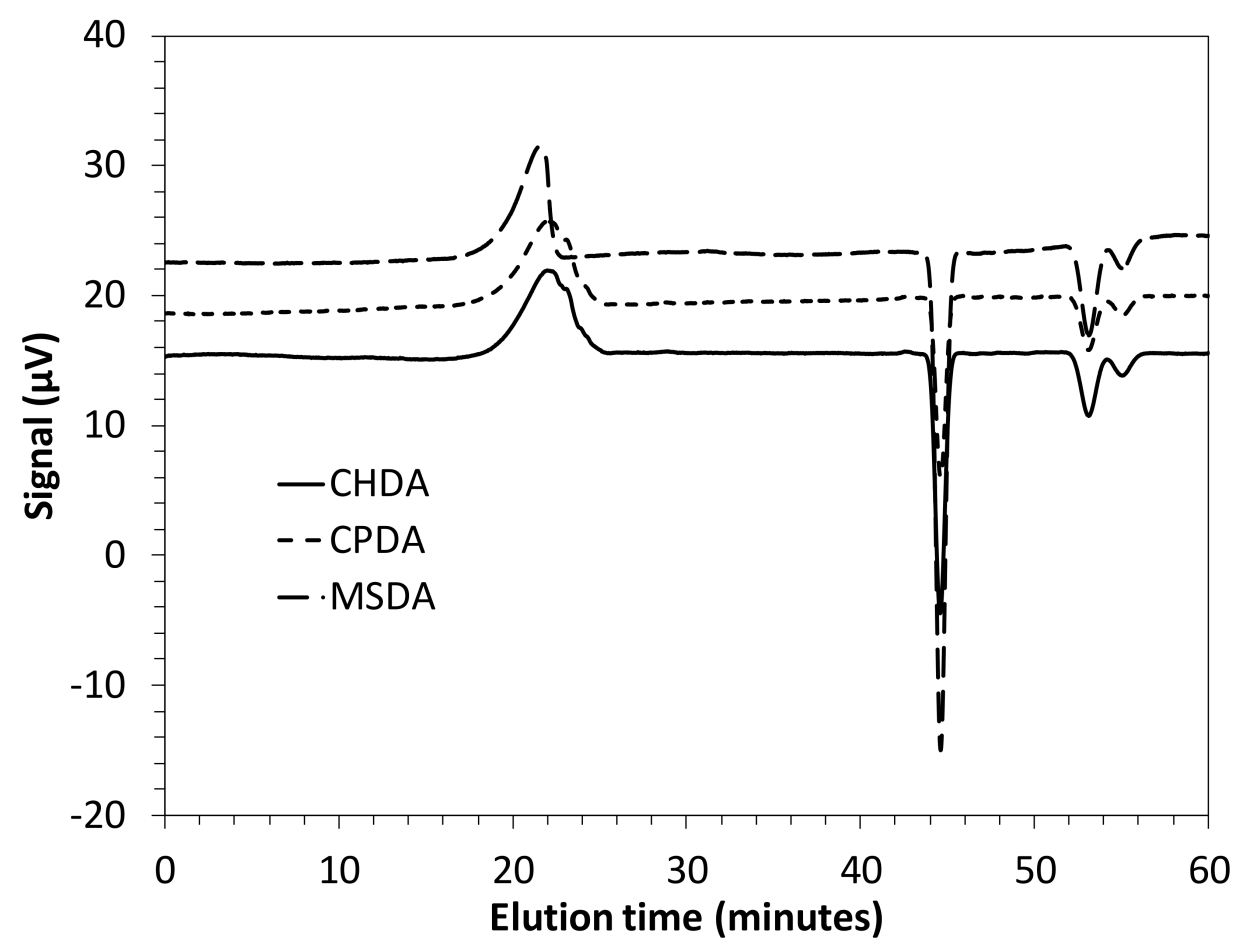
Size exclusion chromatography (SEC) eluographs of the synthesized biopolyimide samples.

The polycondensation reaction was carried out in a 1:1 solution of 4,4’-diamino-α-truxillic dimethyl ester (4ATA ester) and dianhydride in the super dehydrated DMAc, as shown in the Figure 1 [21]. The development of viscosity with the progression of the reaction indicated the formation of poly(amic acid) (PAA, Figure S5). FT-IR analysis of the PAA fibrils showed a broad signal at 2600–3600 cm^−1^ (O–H, stretching), two sharp absorption bands at 1720 cm^−1^ (C–O stretching, carboxylic and ester), and 1670 cm^−1^ (C–O stretching, amide) and aromatic peaks at 1525 and 1432 cm^−1^ (C–H first overtone, aromatic) (Figure S5). The chemical structure of the PI was confirmed by the FT-IR. A signal at 1375 and 1175 cm^−1^ (C–N stretching, imide) confirms the formation of imide ring and the imidization under the annealing process. Furthermore, the spectra shows two peaks for the carbonyl at 1785 cm^−1^ (C–O, asymmetric stretching) and 1716 cm^−1^ (C–O symmetric stretching). The peak at 1211 cm^−1^ was assigned to the C–O (stretch, COOH, COOCH_3_). The number average molecular weight (*M*_n_) and PDI of the PAA was measured by GPC (Figure 2), which were found to be 4.7–7.9 × 10^5^ and 1.4 respectively. 

The biopolyimide crystallinity was investigated by X-ray diffraction (XRD, Figure S7). The XRD pattern of biopolyimide yields two diffraction peaks at 2θ = 6° and 17° overlapping with a broad amorphous halo. The XRD diagram of neat biopolyimide indicates a partial crystallization (19–28%). It is noteworthy to mention the broad halo-midpoint shift towards higher 2θ with the decrease in the dianhydride ring strain. However, the shift in the 2θ from CHDA to MSDA was easily visible maybe due to the complete absence of dianhydride ring strain leading to the flexible amorphous biopolyimide structures. 

The synthesized biopolyimides were thermally stable with 10% degradation (*T*_d10_, Table 1, Figure S8) from 375–397 °C. Glass-transition temperature (*T*_g_, Table 1, Figure S9) of the biopolyimide was determined by DSC. It was observed that the biopolyimide *T*_g_ was dependent upon multiple factors such as molecular weight, ring strain etc. Within the aliphatic ring containing dianhydride containing biopolyimide, the decrease in ring strain was found to be associated with lower *T*_g_. The biopolyimide films were subjected to UV-Vis spectroscopy to understand their optical properties (Table 1 and Figure S10). CPDA based biopolyimide film was too brittle to be cast as a film for UV-Vis measurement. It was observed that the MSDA based biopolyimide shows greater transparency than the CHDA type. However, it was interesting to observe that these biopolyimide films were able to completely block the severe UV-C radiation. 

Biopolyimide’s solubility was verified in a wide range of solvents (polar protic, polar aprotic, non-polar) as represented in Table 1. It was found that all the biopolyimides exhibit very high chemical resistance against non-polar solvents. On the contrary, all the biopolyimides were soluble in strong acids such as sulfuric acid or trifluoracetic acid. Moreover, in the case of aprotic solvents it was found that the MSDA, CPDA, and CHDA based biopolyimides show high solubility, especially with NMP, DMF, and DMSO. It is noteworthy to mention that all previous biopolyimides with 4ATA in the literature were insoluble in all aprotic solvents. Therefore, this article is the first report of the syntheses of organo-soluble biopolyimides without introducing any bulkier side chain or conventional strategies.

(1) $$\varphi^{2}=\frac{K\times \left(n+1\right)}{MW}\times \left|\frac{Torsion\;Energy}{Polar\;Surface\;Area}\right|+\frac{C}{MW}$$

**Figure 3:**
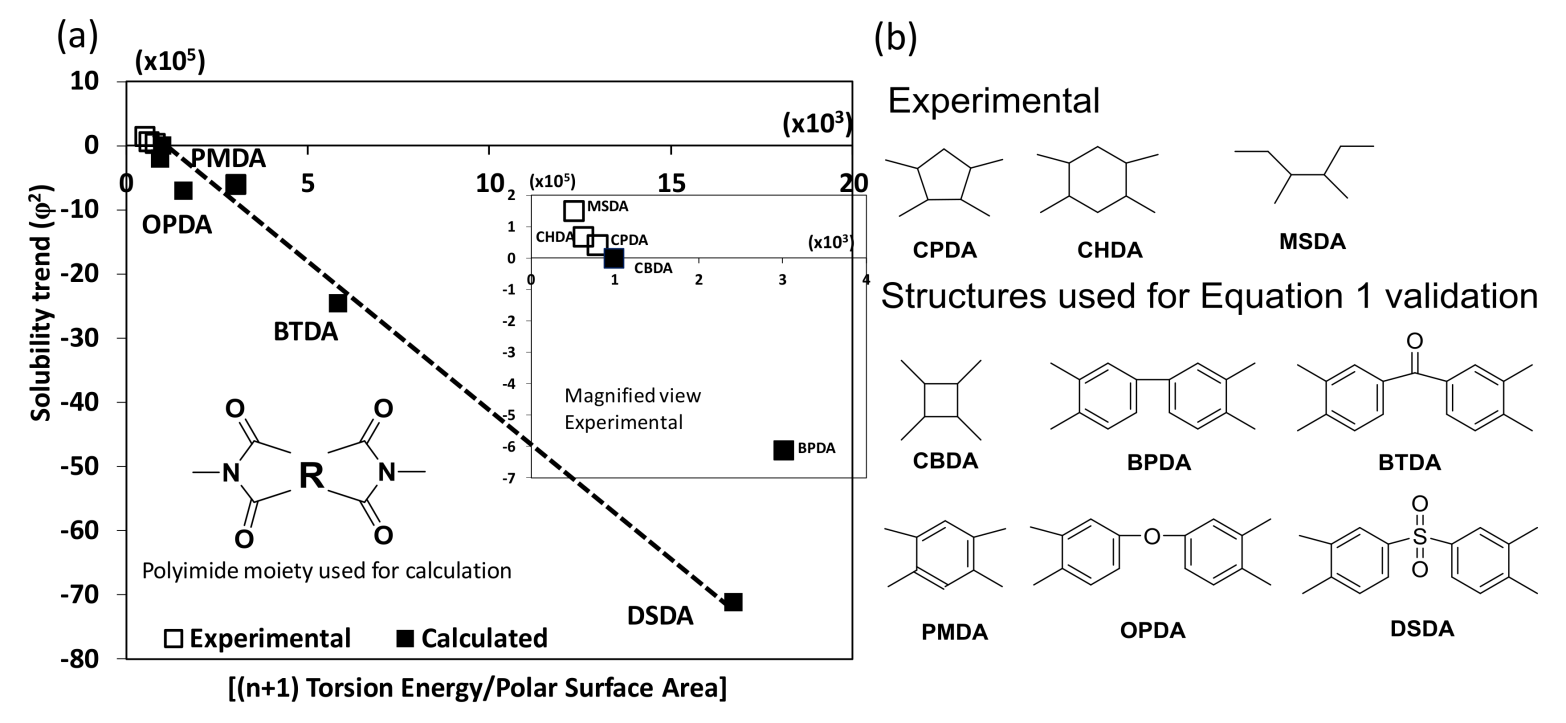
(**a**) Biopolyimide structure-solubility relationship. The calculated test samples were utilized for the validation of the solubility trend. Positive values of *φ*^2^ represent soluble biopolyimide, while the negative values indicate insoluble biopolyimide. (**b**) The structures of “R” for various dianhydrides are shown for the experimental and calculated biopolyimide units.

It was observed that the polymer with more relaxed backbone structure solubulizes faster in the aprotic solvents. In other words, maybe the torsion strain in the polymer backbone plays an important role in determining the solubility trend for the aromatic polyimide [23]. In general, the solubility parameter (δ) of the material may be explained using the Hildebrand-Scatchard solution theory, which is defined as the square root of the energy required to make infinite separation between the polymer chains [24]. Hildebrand-Scatchard solution theory describes several kinds of cohesion forces responsible for holding the polymer chains but does not consider the repulsion forces [25,26,27,28], which are considered to complicate the situation and present a challenge for establishing the polymer structure solubility relationship [29,30]. At the molecular level the intra-chain movability decides the infinite separation between the chains to yield the soluble polymer. The movability of the polymer chain may be correlated with the torsion energy of the repetitive unit. Furthermore, the localized polar surface area of the repetitive unit interacts (attractive/repulsion) with the solvent and controls the solvent diffusion inside the polymer particle. Moreover, the cohesive energy (inter-/intra-molecular) between the molecules is also important to ascribe for the ease of separating the individual molecules from each other. The greater cohesive energy would make it harder to separate the molecules and thereby show lower solubility [31]. Considering these solubility triggering parameters, an equation (Equation (1)) was developed for determining the trend of the 4ATA ester based aromatic biopolyimide solubility through a parameter *φ*, dimensionally equivalent to the conventional solubility parameter (δ). The solubility test of the biopolyimide derived from 4ATA ester and various kinds of dianhydride was performed at room temperature (298 K) in NMP. As exprssed in Equation (1), *MW* is the polymer weight-average molecular weight, *n* is the number of aromatic carbon atoms, *C* is a constant dependent on the solvent and/or kind of diamine in the biopolyimide and *K* is the solubility inducing constant determined by a data fitting method. The physical significance of the solubility inducing constant is reported in the Supplementary Information. The biopolyimide solubility trend results were found to show a linear relationship between the solubility parameter and the ratio of torsion energy to the polar surface area, as shown in Figure 3. The positive value of *φ*; denotes polymer solubility in NMP. The negative value of the solubility parameter corresponds to the insoluble biopolyimide because of an imaginary number.

The applicability of Equation (1) was verified by the random dianhydride selection. CPDA and BPDA were selected as an aliphatic and aromatic dianhydride, repectively. Biopolyimides were prepared by the already stated methodology. The biopolyimide was subjected to the solubility test in various solvents and it was found that the CPDA exhibited similar solubility characteristics as compared to the CHDA albeit slightly slower. On the contrary, the the 4ATA-ester based biopolyimides comprising various dianhydrides in the literature such as, CBDA, BTDA, OPDA, DSDA, PMDA, and BPDA were not soluble in any of the conventional solvents, except strong acids (sulfuric acid). The torsion energy and the polar surface areas of the dianhydride repetitive units were determined and subjected for validation of Equation (1). The results were surprisingly well correlated with a regression coefficient of 0.995%. This study exemplifies not only several 4ATA based organo-soluble biopolyimides but also a methodology to determine the solubility trend for the same. This approach may be utilized for predicting polymer solubility to yield better control over the synthesized polymers.

## 4. Conclusions

This research has witnessed the syntheses of several new biopolyimides using 4ACA derived diamine and various dianhydrides with varying degrees of molecular torsion energy. Several biopolyimides show solubility in conventional aprotic organic solvents. A systematic investigation was conducted to establish a relationship between polyimide solubility and structural features by varying only the kind of dianhydride. It was observed that the incorporation of the molecular repetitive units with lower torsion energy favors biopolyimide solubility. Lower ring strain incorporated in the biopolyimide repetitive units through various dianhydride structures is accompanied a lower glass transition temperature due to more flexible polymer microstructures. These properties make the synthesized biopolyimides attractive materials for practical applications demanding processable high-performance engineering plastics. Furthermore, this research gives an insight into the prediction, design, and synthesis of more advanced soluble plastics.

## Figures and Tables

**Scheme 1:**
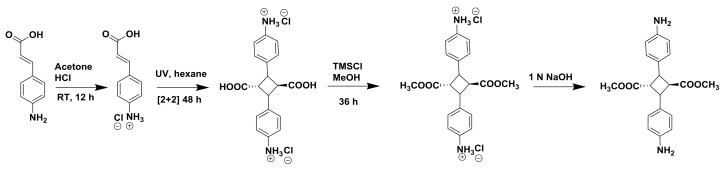
Monomer synthesis from 4-aminocinnamic acid (4ACA).

**Table 1:** Biopolyimide optical, thermal, and solubility characteristics.

Polyimide	CHDA	MSDA	CPDA
Dianhydride			
*T*_d10_^a^ (°C)	397	375	391
*T*_g_^b^ (°C)	259	273	294
*T* ^c^ (%)	51	57	ND
*M*_n_^d^ (g/mol)	4.8 × 10^5^	7.8 × 10^5^	5.9 × 10^5^
*M*_w_/*M*_n_^d^	1.4	1.4	1.3
Solubility Test			
Hexane	-	-	-
Toluene	-	-	-
Dichloromethane	-	-	-
Chloroform	-	-	-
Diethylether	-	-	-
Water	-	-	-
Methanol	-	-	-
Ethanol	-	-	-
Acetone	-	-	-
Acetonitrile	-	-	-
Ethylacetate	-	-	-
Tetrahydrofuran	±	±	±
*N*-Methyl-2-pyrrolidone	+	+	+
Dimethyl sulfoxide	+	+	+
*N*,*N*-dimethylformamide	+	+	+
*N*,*N*-dimethylacetamide	+	+	+
Conc. H_2_SO_4_		+	+

^a^ *T*_d10_ represents the 10 wt % thermal degradation temperature measured under nitrogen through thermogravimetric analysis (TGA). ^b^ *T*_g_ represents the glass transition temperature measured using differential scanning calorimetry (DSC). ^c^ *T* shows the transmittance at *λ* = 450 nm measured by UV-Vis spectroscopy. ^d^ *M*_n_ and *M*_w_/*M*_n_ represent the number-average molecular weight and polydispersity index of the biopolyimide measured by gel permeation chromatography. ND represents not determined.

## Supplementary Materials

The following are available online at http://www.mdpi.com/2073-4360/10/4/368/s1. Figure S1: ^1^H NMR characterization for 4ACA salt; Figure S2: ^1^H NMR characterization for 4ATA salt; Figure S3: ^1^H NMR characterization for 4ATA methyl ester salt; Figure S4: ^1^H NMR characterization for 4ATA methyl ester; Figure S5: FTIR characterization peak assignments for the prepared poly(amic acid) and polyimide; Figure S6: FTIR characterization for the prepared polyimide with characteristic peaks shown in vertical bands; Figure S7: WAXD pattern for the synthesized biopolyimides; Figure S8: TGA curves for the synthesized biopolyimides; Figure S9: DSC curves for the biopolyimides; Figure S10: Optical transparency of the synthesized biopolyimides; Biopolyimide Solubility Equation see [32].
