# Supplementary material for: Molecular Design of Soluble Biopolyimide with High Rigidity
Source: Polymers (Basel). 2018 Mar 26;10(4):368. doi: 10.3390/polym10040368 (PMC6414993; doi:10.3390/polym10040368)
Supplement: Supplementary file 1 [file polymers-10-00368-s001.pdf]

Supporting Information

Molecular Design of Soluble Biopolyimide with High Rigidity

Sumant Dwivedi [1] and Tatsuo Kaneko [1,2,*]

[1] Graduate School of Advanced Science and Technology, Energy and Environment Area, Japan Advanced Institute of Science and Technology, 1-1 Asahidai, Nomi, Ishikawa 923-1292, Japan; supernalsumant@gmail.com
[2] Japan Science and Technology, ALCA, Tokyo 102-0076, Japan
* Correspondence: kaneko@jaist.ac.jp

Monomer Syntheses. 4,4′-Diamino-α-truxillic acid (4ATA) dihydrochloride was synthesized by the drop-wise addition of 12 N hydrochloric acid solution (5.6 ml) in a solution of 4ACA (2.0 g, 12.4 mmol) in acetone (30 ml) to produce 4-aminocinnamic acid hydrochloride (1.74 g, 4.35 mmol). The obtained product was subjected to irradiation by a 100-W high pressure Hg-lamp (Omni Cure S1000, EXFO Photonic Solution Inc.) with a 250−450 nm band-pass filter with an intensity of 2.7 mW/cm^3 for 24−36 h to induce [2 + 2] photocycloaddition. The reaction was monitored by ^{1}H NMR (400 MHz, DMSO-d^6, δ, ppm) by disappearance of peaks for the olefinic protons: 3.82 (dd, 2H, J = 7.7, 9.6 Hz), 4.30 (dd, 2H, J = 7.7, 9.6 Hz), 7.33 (d, 4H, J = 7.7 Hz), 7.45 (d, 4H, J = 7.7 Hz), 10.37 (s, 6H), 12.07 (s, 2H).

The obtained 4,4′-diamino-α-truxillic acid (4ATA) dihydrochloride was subjected to esterification. A solution of 4 ATA (1.71 g, 4.27 mmol) in methanol was prepared and trimethylsilyl chloride (TMSCl) was added drop-wise. The reaction was monitored by ^{1}H NMR (400 MHz, DMSO-d^6, δ, ppm) and allowed to take place for 12 h. The obtained product was filtered and dried at 40 °C under vacuum for 6 h. The dimethyl ester of 4ATA salt (1.83 g, 4.28 mmol) was dissolved in water and neutralized by 1 N NaOH solution to obtain 4,4′-diamino-α-

truxillic dimethyl ester (1.29 g, 3.64 mmol). The obtained product was dried and subjected to soxhlet purification using ethyl acetate to obtain crystals. The purified 4,4′-diamino-α-truxillic dimethyl ester was confirmed using ^{1}H NMR (400 MHz, DMSO-d^6, δ, ppm).

The crystals of 4,4′-diamino-α-truxillic dimethyl ester, (0.20 g, 0.5647 mmol) were dissolved in DMAc (0.8 mL, 9.6 mmol) under a nitrogen atmosphere followed by the addition of CHDA (0.13 g, 0.5647 mmol). The reaction mixture was stirred at room temperature to produce a clear to pale yellow viscous poly(amic acid) (PAA) solution in 48 h. The PAA solution was diluted in DMAc, and then added dropwise in methanol/water mixture to precipitate PAA fibers, which were filtered and dried under vacuum at 60 °C for 12 h. The PAA film was obtained by casting a PAA homogeneous solution in DMAc onto a glass plate and heating at 75 °C. The obtained PAA films were subjected to thermal imidization in an oven under reduced pressure by stepwise heating at 100, 150, 200, and 250 °C for 1 h at each step to obtain polyimide films. A similar procedure for the polyimide preparation was followed with other dianhydride structures.

Polyimide Synthesis and Characterization. Production of diamine from 4ACA demands the amalgamation of two precursor units. A [2+2] photocycloaddition reaction of *trans*-cinnamic acid, with radiations (λ >260 nm) to yield α-truxillic acid. Olefinic functionality of 4ACA was utilized to obtain the corresponding α-truxillic acid from the 4ACA salt, characterized by ^{1}H NMR (Figure S1). The reaction was monitored by ^{1}H NMR with the disappearance of the olefinic protons (δ = 7.44−7.40 and 6.15−6.12) and development of cyclobutane proton signals (δ = 4.36–3.80) (Figure S2). Precise monomer design demands the protection of the two-carboxylic acid groups of 4ATA to avoid any possible interference during the polymerization. Therefore, esterification of 4ATA was performed with methanol and TMSCl,

confirmed by ^{1}H NMR (Figure S3). Finally, the 4ATA-methyl ester salt was neutralized by 1M NaOH aqueous solution and the final product was confirmed by ^{1}H NMR (Figure S4).

The polycondensation was carried out in a 1:1 solution of 4ATA ester and various dianhydrides in super dehydrated DMAc (Figure S5). The development of viscosity with the progression of the reaction indicates the formation of poly(amic acid) (PAA). FT-IR analysis of the PAA fibrils shows a broad signal around 2600–3600 cm^{-1} (O−H, stretching), two sharp absorption bands at 1720 cm^{-1} (C−O stretching, carboxylic and ester) and 1670 cm^{-1} (C−O stretching, amide) and aromatic peaks at 1525 cm^{-1} and 1432 cm^{-1} (C−H first overtone, aromatic) (Figure S5,S6). The molecular weight of the PAA was found to be high enough to form a self-standing film. Preparing the PAA films by solution and stepwise annealing yields polyimide films. The chemical structure of the PI was confirmed by the FT-IR. A signal at 1375 cm^{-1} and 1175 cm^{-1} (C−N stretching, imide) confirmed the formation of imide ring and the imidization under the annealing process. Furthermore, the spectra show two peaks for carbonyl at 1785 cm^{-1} (C−O, asymmetric stretching) and 1716 cm^{-1} (C-O symmetric stretching). The obtained polyimide was confirmed to have high chemical resistance except for concentrated sulfuric acid.

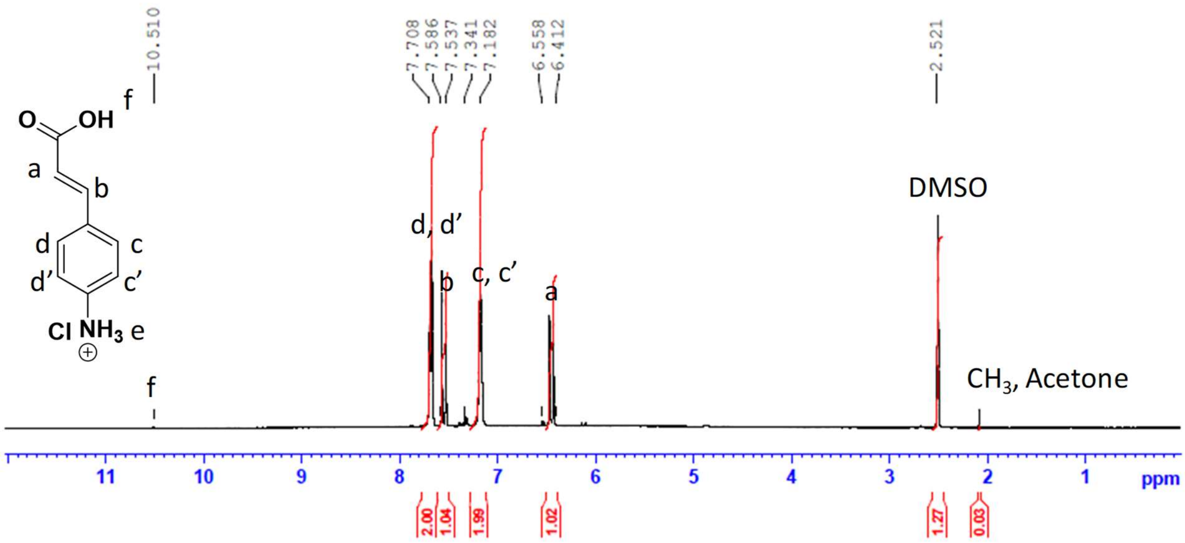

Figure S1 ^{1}H NMR characterization for 4ACA salt.

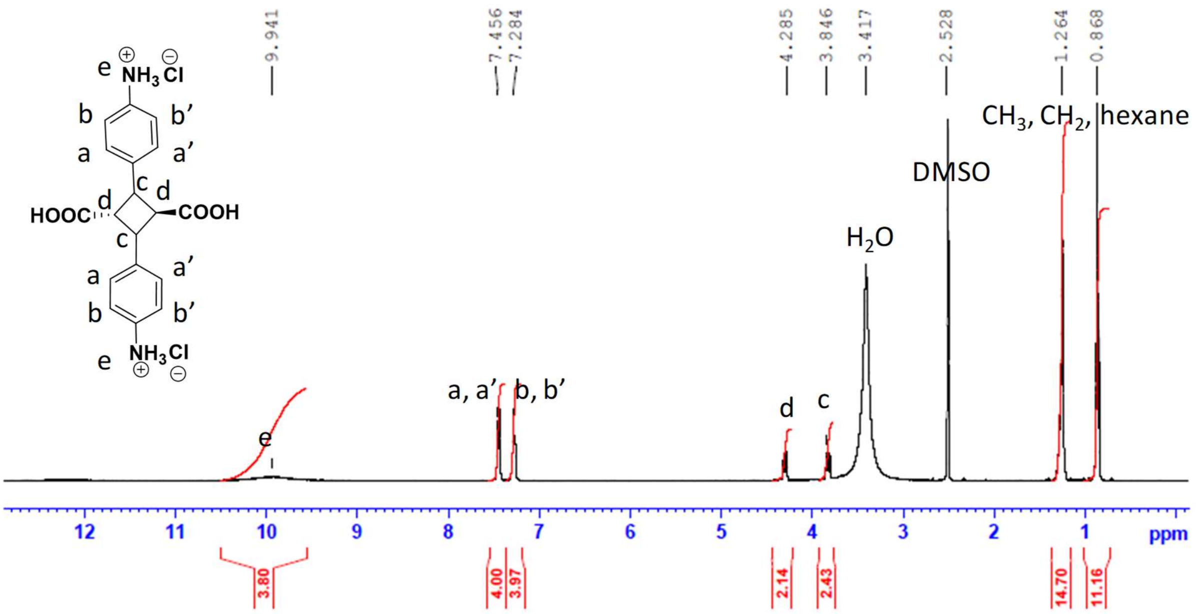

Figure S2 ^1H NMR characterization for 4ATA salt.

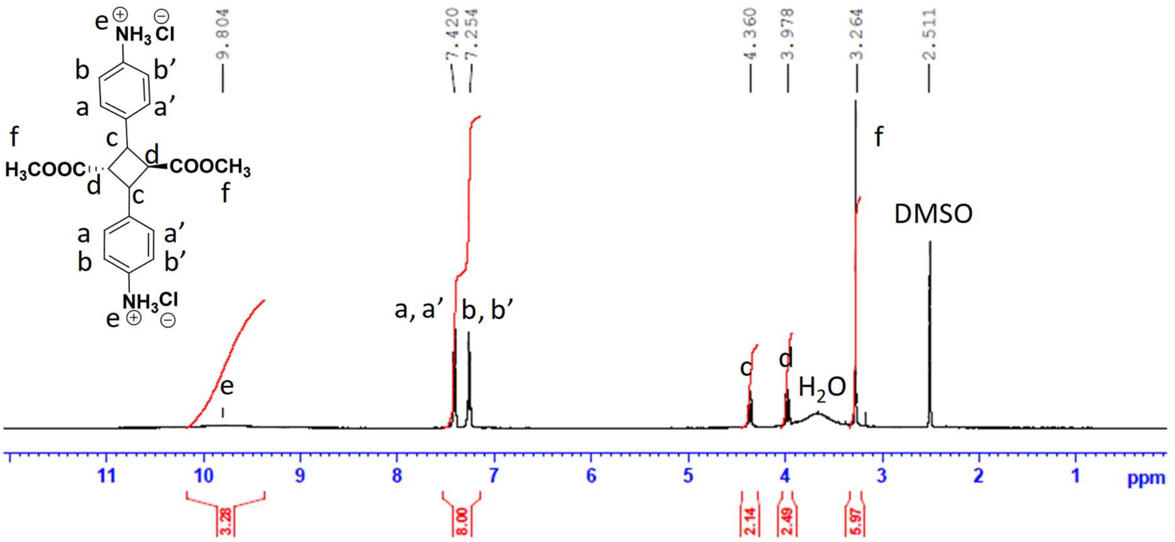

Figure S3 ^1H NMR characterization for 4ATA methyl ester salt.

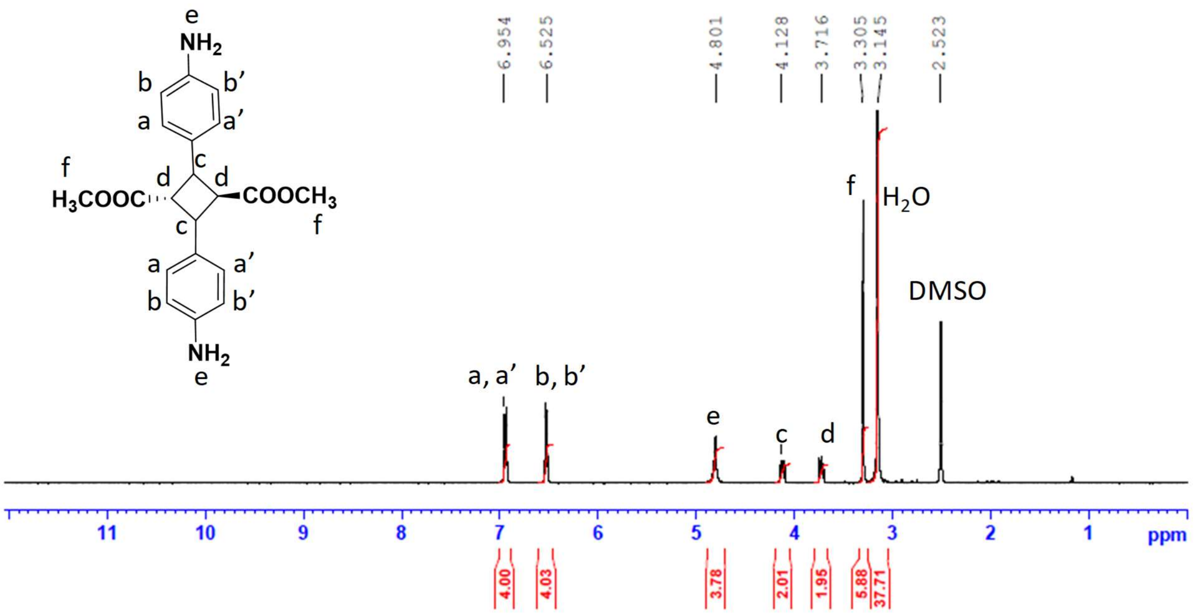

Figure S4 ^{1}H NMR characterization for 4ATA methyl ester.

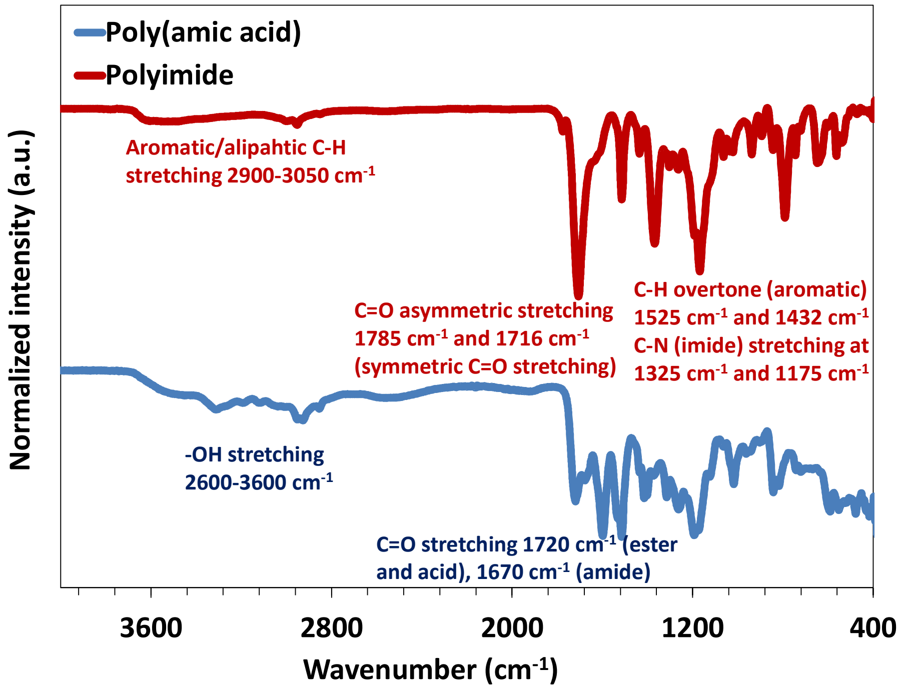

Figure S5 FTIR characterization peak assignments for the prepared poly(amic acid) and polyimide.

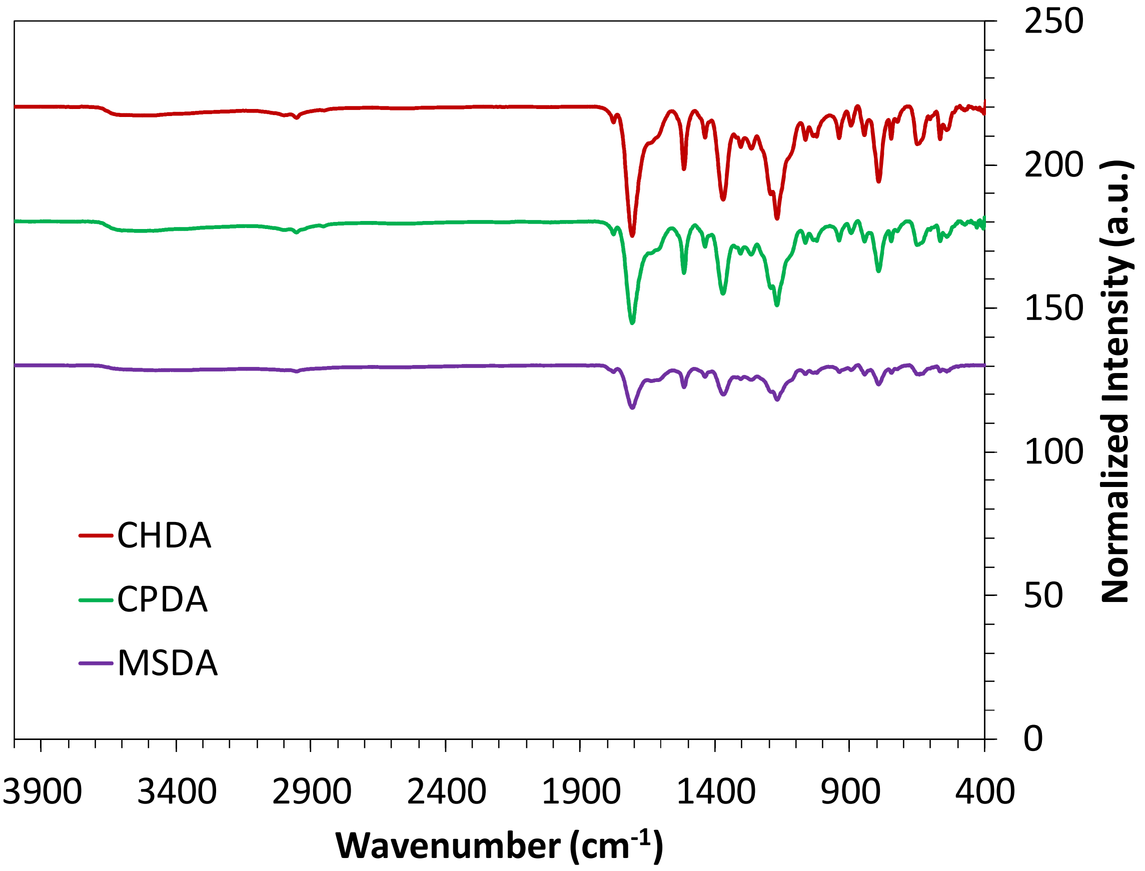

Figure S6. FTIR characterization for the prepared polyimide with characteristic peaks shown in vertical bands.

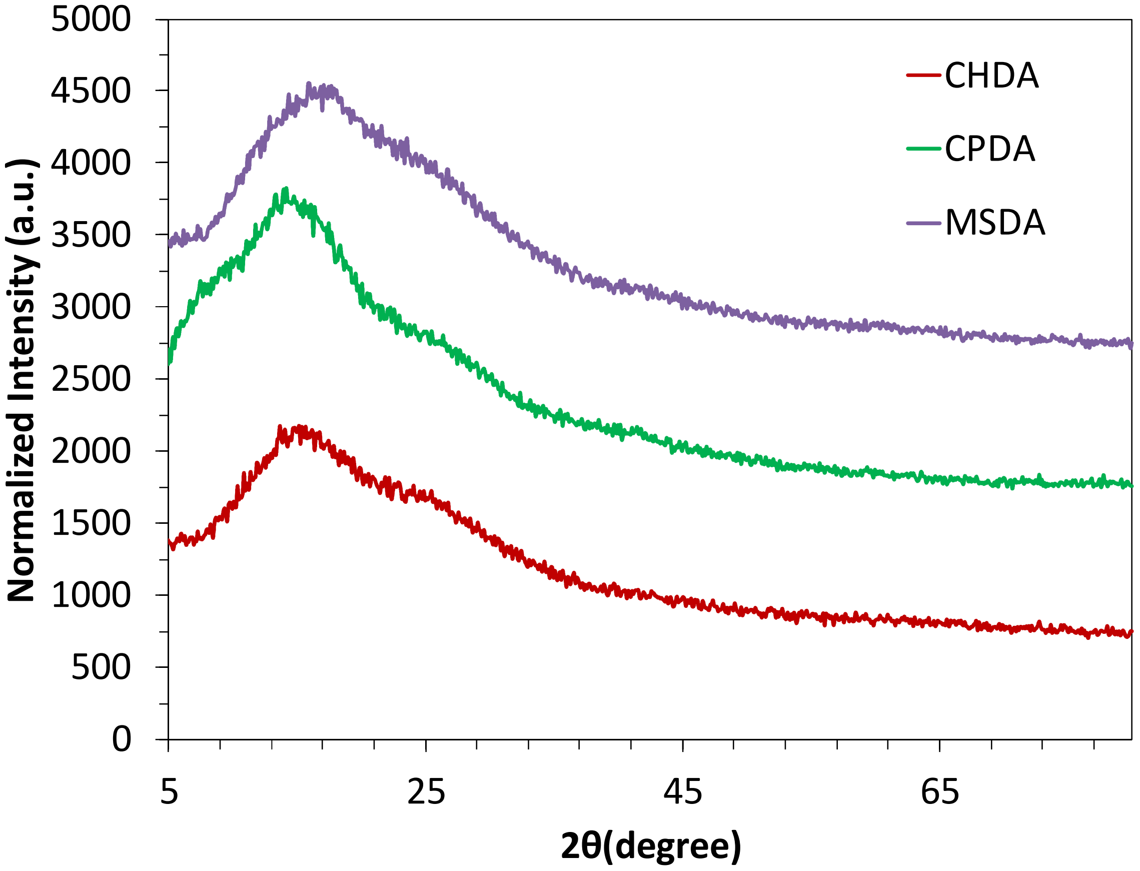

Figure S7 WAXD pattern for the synthesized biopolyimides.

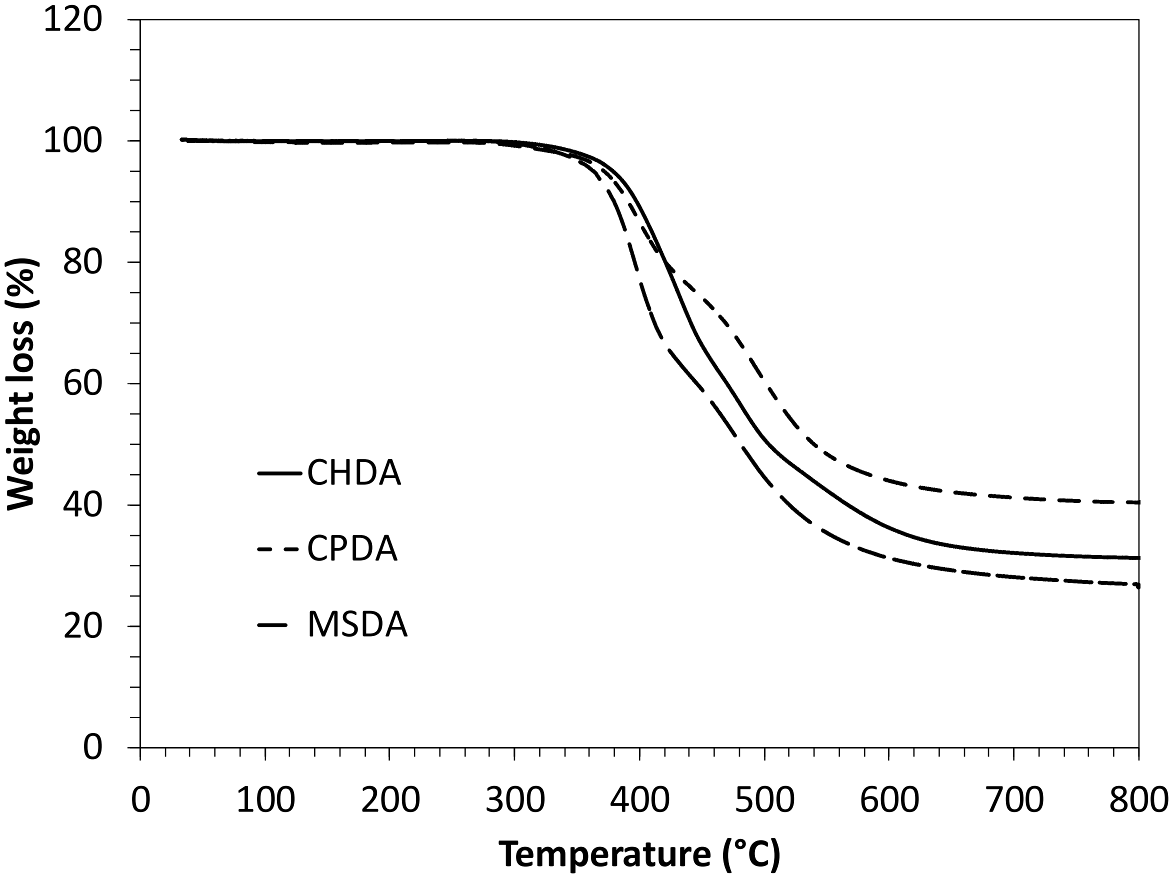

Figure S8 TGA curves for the synthesized biopolyimides.

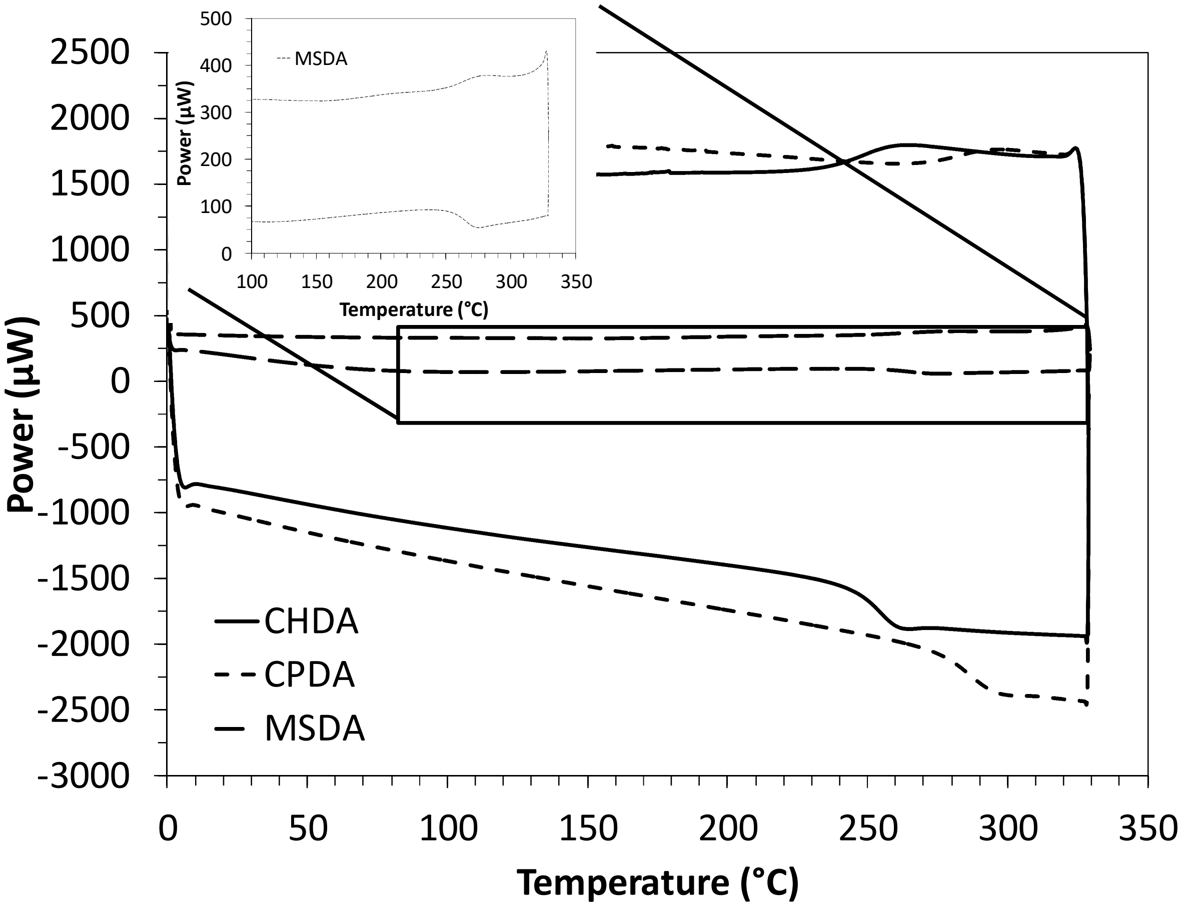

Figure S9 DSC curves for the biopolyimides.

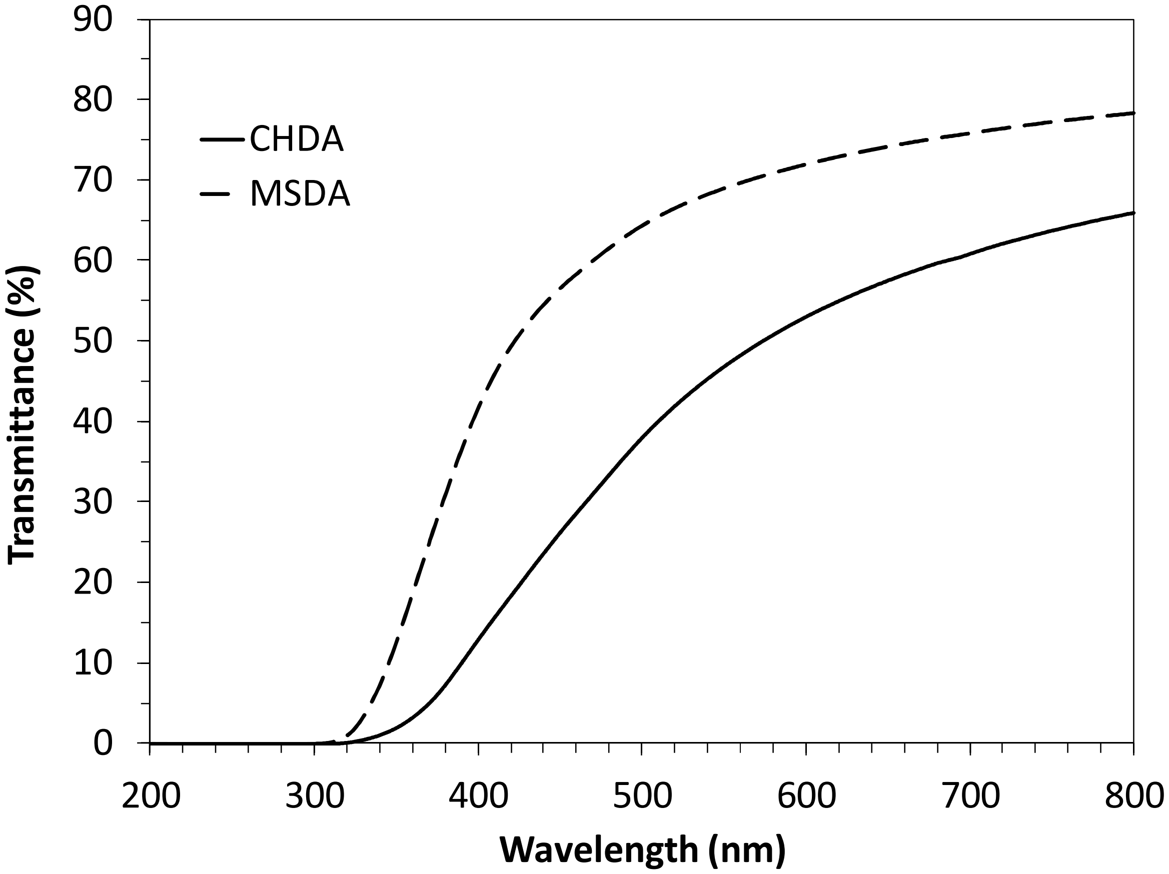

Figure S10 Optical transparency of the synthesized biopolyimides

Biopolyimide Solubility Equation

$$\varphi^2 = \frac{K \times (n+1)}{MW} \times \left|\frac{Torsion\ Energy}{Polar\ Surface\ Area}\right| + \frac{C}{MW}$$

$$K = -42.651 \times \left[\left(\sqrt{n^2+1}\right) - m\right]$$

$$C = 40587$$

φ represents the solubility trend parameter, MW is weight average molecular weight of the polymer, C is a constant, n are the number of aromatic carbon atoms and, m are the number of linking atoms between two dianhydride units. The value of K was determined by the best fit method [32].

32 .Moore. D.; McCabe. G. Introduction to the Practice of Statistics. W. H. Freeman and Co., London **2003**.
